# Recognizing brain activities by functional near-infrared spectroscope signal analysis

**DOI:** 10.1186/1753-4631-2-3

**Published:** 2008-07-01

**Authors:** Truong Quang Dang Khoa, Masahiro Nakagawa

**Affiliations:** 1 Chaos and Fractals Informatics Laboratory, Nagaoka University of Technology, 1603-1 Kamitomiokamachi, Nagaoka, Niigata, 940-2188, Japan

## Abstract

**Background:**

Functional Near-Infrared Spectroscope (fNIRs) is one of the latest technologies which utilize light in the near-infrared range to determine brain activities. Near-infrared technology allows design of safe, portable, wearable, non-invasive and wireless qualities monitoring systems. This indicates that fNIRs signal monitoring of brain hemodynamics can be value in helping to understand brain tasks. In this paper, we present results of fNIRs signal analysis to show that there exist distinct patterns of hemodynamic responses which recognize brain tasks toward developing a Brain-Computer interface.

**Results:**

We applied Higuchi's fractal dimension algorithms to analyse irregular and complex characteristics of fNIRs signals, and then Wavelets transform is used to analysis for preprocessing as signal filters and feature extractions and Neural networks is a module for cognition brain tasks.

**Conclusion:**

Throughout two experiments, we have demonstrated the feasibility of fNIRs analysis to recognize human brain activities.

## Background

Neurophysiological and neuroimaging technologies have contributed much to our understanding of normative brain function. Commonly employed techniques such as electroencephalography (EEG), event-related brain potentials (ERPs), magnetoencephalography (MEG), positron emission tomography (PET), singlepositron emission computed tomography (SPECT), and functional magnetic resonance imagining (fMRI) have dramatically increased our understanding of a broad range of brain activities [1]. EEG and ERP paradigms have contributed important data for developing models of cognitive and emotional processing. However, EEG measures are limited in their ability to provide the precise location of an electrical source. EEG does yield spatial information, but this spatial information must be reconstructed by probabilistic models. fMRI is currently considered the "gold standard" for measuring functional brain activation. The limitations of fMRI relative to fNIRs include the fact that participants must lie within the confines of the magnet bore, which limits its use for many applications. fMRI is also highly sensitive to movement artifact; subject movements on the order of a few millimeters can invalidate the data. Finally, fMRI systems are quite expensive [1].

In recent years, functional near-infrared spectroscopy (fNIRs) has been introduced as a new neuroimaging modality with which to conduct functional brain-imaging studies. fNIRs technology uses specific wavelengths of light, introduced at the scalp, to enable the noninvasive measurement of changes of deoxygenated hemoglobin (deoxy-Hb) and oxygenated hemoglobin (oxy-Hb) during brain activity. Wireless fNIRs system consists of personal digital assistant (PDA) software controlling the sensor circuitry, reading, saving, and sending the data via a wireless network. This technology allows the design of portable, safe, affordable, noninvasive, and minimally intrusive monitoring systems [2].

For such advanced features, fNIRs signal processing really becomes an attractive field for computational science. In [3], M. Izzetoglu et al. investigated canceling motion artifact noise from fNISs signals by Wiener filter. The authors indicated that the noise including in fNIRs is an important limitation on the use of optical data in these applications. Motion artifact can cause the NIR detectors to shift and lose contact with the skin, exposing them to either ambient light or to light emitted directly from the NIR sources or reflected from the skin, rather than being reflected from tissue in regions of interest. Hence, canceling noise from fNIRs signals is one of necessary tasks in order to use fNIRs as a brain monitoring technology in its full potential to many real life application areas. In [4], M. Izzetoglu et al. presented statistical analysis of fNIRs signals for the purpose of cognitive state assessment while the user performs a complex task. The results indicated that the rate of change in blood oxygenation of fNIRs signals was significantly sensitive to task load changes and correlated fairly well with performance variables. In [5,6], S. Fantini et al. describe a specific frequency-domain instrument for near-infrared spectroscopy and imaging of tissues that shows the hemodynamic changes monitored with NIR spectroscopy correlate with the activation state of the cortex in response to a stimulus. They investigated the possibility of combining phase and average intensity data in fNIRs frequency-domain imaging of the brain activation presenting different spatial/temporal features.

In [7], R. Sitaram et al. presented results of signal analysis indicating that there exist distinct patterns of hemodynamic responses which could be utilized in a pattern classifier. The fNIRs signals were processed to remove artifacts from heart beat and high frequency noise from muscle activities by Chebyshev type II filter. And then, they applied two different pattern recognition algorithms separately, Support Vector Machines (SVM) and Hidden Markov Model (HMM), to classify the data offline. SVM classified with an average accuracy of 73%, while HMM performed better with an average accuracy of 89%.

In this work, we consider fNIRs signals and analyze irregular and complex characteristics by Higuchi fractal dimension algorithms [10]. This method was successfully applied for EEG bio-signal processing in [8,9]. Fractal dimension values along period of time serve as meaningful characteristics of studied bio-signals. With obtained experiment results, fractal dimensions of fNIRs signals can not clearly indicate information of brain activities. Therefore, we proposes Wavelet-Neuron model to recognize brain activities through fNIRs signals. Wavelet transform became the foundation for the most popular techniques for signal analysis and representation in a wide range of applications. Wavelets processing play a role of extraction algorithm to draw features of fNIRs signals and to filter high frequency noises. Extracted features are inputs of neural networks to classify brain tasks. Neural networks are very powerful tools for pattern recognition. The neural network used wavelets coefficients as its inputs and brain activities are depicted by outputs. The paper is organized as follows: In section 2, the mathematics basic models are set up including Higuchi's fractal dimension algorithm, Wavelet transform, and Neural Network model. In section 3, fNIRs data acquisition is described including instruments and 2 experiments. Section 4 shows results and discussion. Section 5 is conclusion.

## Methods

### Higuchi fractal dimension

Higuchi's algorithm shown in [10] performs fractal dimension of a time series directly in the time domain. Its principle is based on a measure of length, L(k), of the curve that represents the considered time series while using a segment of k samples as a unit. If L(k) scales like

(1) $$\text{L}(\text{k})\approx {\text{k}}^{-{\text{D}}_{\text{f}}}$$

Fractal dimension, D_f_, equals 1 for a simple curve and equals 2 for a curve which nearly fills out the whole plane. D_f _measures complexity and irregular characteristics of time series signals.

From a given time series: X(1), X(2), ..., X(N) the algorithm constructs k new time series:

$${\text{X}}_{\text{m}}^{\text{k}}:\text{X}(\text{m}),\text{X}(\text{m}+\text{k}),\text{X}(\text{m}+2\text{k}),...,\text{X}(\text{m}+\mathrm{int}((\text{N-m})/\text{k})*\text{k})\text{ for m}=1,2,...,\text{k}$$

where:

N – total number of samples,

m – initial time,

k – interval time,

int (r) – integer part of a real number r, set $\text{M}=\mathrm{int}\left(\frac{\text{N}-\text{m}}{\text{K}}\right)$

The length, L_m_(k), of each curve ${\text{X}}_{\text{m}}^{\text{k}}$

(2) $${\text{L}}_{\text{m}}(\text{k})=\frac{1}{\text{k}}\left[\left(\sum_{\text{i}=1}^{\text{M}}\left|\text{X}(\text{m}+\text{i}*\text{k})-\text{X}(\text{m}+(\text{i}-1)*\text{k)}\right|\right)\frac{\text{N}-1}{\text{M}*\text{k}}\right]$$

where N – total number of samples.

L_m_(k) is not 'length' in Euclidean sense, it represents the normalized sum of absolute values of difference in ordinates of pair of points distant k (with initial point m). The length of curve for the time interval k, L(k), is calculated as the mean of the k values L_m_(k) for m = 1, 2,..., k

(3) $$\text{L}(\text{k})=\frac{\sum_{\text{m}=1}^{\text{k}}{\text{L}}_{\text{m}}(\text{k})}{\text{k}}$$

The value of fractal dimension, D_f_, is calculated by a least-squares linear best-fitting procedure as the slope coefficient of the linear regression of the log-log graph of (1)

### Wavelet transform

Most interesting signals contain numerous nonstationary or transitory characteristics: drift, trends, abrupt changes, and beginnings and ends of events. These characteristics are often the most important part of the signal, and Wavelets analysis is well suited to detecting them.

From [11], the wavelets transform of a signal s is the family C(a,b), which depends on two indices a and b. C represents how closely correlated the wavelet is with this section of the signal. The higher C is, the more the similarity. More precisely, if the signal energy and the wavelet energy are equal to one, C may be interpreted as a correlation coefficient. The set to which a and b belong:

(4) $$\text{C}(\text{a},\text{b})=\int_{\text{R}}\text{s}(\text{t})\frac{1}{\sqrt{\text{a}}}\psi (\frac{\text{t}-\text{b}}{\text{a}})\text{dt}$$

Where:

a = 2^j^, b = k2^j^, (j,k) ∈ Z^2^

ψ is wavelet functions

a is scale of wavelets functions

b is position of wavelets functions on the signal s.

From an intuitive point of view, the wavelets decomposition consists of calculating a "resemblance index" between the signal and the wavelets located at position b and of scale a. If the index is large, the resemblance is strong, otherwise it is slight. The indexes C(a,b) are called coefficients.

Let us fix j and sum on k. A detail D_j_(t) is nothing more than the function

(5) $${\text{D}}_{\text{j}}(\text{t})=\sum_{\text{k}\in \text{Z}}\text{C}(\text{j},\text{k})\psi_{\text{j},\text{k}}(\text{t})$$

Now, let us sum on j. The signal is the sum of all the details:

(6) $$\text{s}=\sum_{\text{j}\in \text{z}}{\text{D}}_{\text{j}}$$

The details have just been defined. Take a reference level called J. There are two sorts of details. Those associated with indices j<J correspond to the scales a = 2^j ^≤ 2J which are the fine details. The others, which correspond to j > J, are the coarser details. We group these latter details into:

(7) $${\text{A}}_{\text{J}}=\sum_{\text{j}>\text{J}}{\text{D}}_{\text{j}}$$

which defines what is called an approximation of the signal s. We have just created the details and an approximation.

(8) $$\text{s}={\text{A}}_{\text{J}}+\sum_{\text{j}\leq \text{J}}{\text{D}}_{\text{j}}$$

The equality signifies that s is the sum of its approximation A_j _and of its fine details. From the previous formula, it is obvious that the approximations are related to one another by

(9) A_j-1 _= A_j _+ D_j _

**Figure 1:**
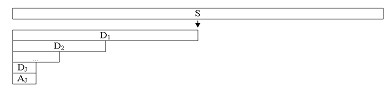
Wavelets transform of a sequence S, with D as details, A as an approximation, and J as level of wavelets analysis.

The total number of computed coefficients in the matrix shown in Fig. 1 is precisely equal to the length of the original sequence s. A_j _depicts as feature vectors that serve as input patterns of neural networks in next subsection.

To quantify the improvement, the change in signal to noise ratio (SNR) was used as a measurement of performance. The SNR-gain is computed as

(10) $${\text{SNR}}_{\text{gain}}=10\log_{10}\frac{{\text{AvgPower}}_{\text{rawSignal}}}{{\text{AvgPower}}_{\text{filteredSignal}}}$$

The average power for the raw and filtered signals, by Parseval's theorem, is computed by Power Spectral Density (PSD) as

(11) $$\text{AvgPower}=\frac{1}{2\pi }\sum_{-\pi }^{\pi }\text{PSD}(\omega )$$

### Neural Networks

The main aim of this paper is recognition and classification fNIRs signals corresponding to brain activities. After testing for non-linear in fNIRs signal by Higuchi fractal dimension and feature extracting by wavelet transforms, neural networks are very powerful tools for classification or pattern recognition shown in [11]. Informative features are extracted from the coefficients computed with the wavelets transform and used as inputs for classification.

**Figure 2:**
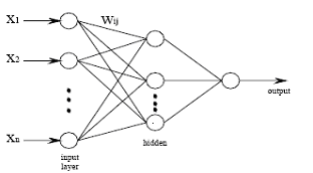
Multi-layer feed forward neural network for recognizing brain activities.

The multi-layer fully connected feed-forward neural network depicted in Fig. 2 is used here; it includes an input layer, one hidden layer and an output layer. Signal propagation is allowed only from the input layer to the hidden layer and from the hidden layer to the output layer. Input variables come from A_J_, wavelets coefficients, mentioned above section. The outputs are the desired classes. The number of inputs is the number of channels, and the number of hidden nodes, transfer functions affect the training performance hence need to be chosen carefully.

As usual, the back propagation training is based on the minimization of the following quadratic cost function:

(12) $$\text{E}=\frac{1}{2}\sum_{\text{n}=1}^{\text{N}}{\left({\text{y}}_{\text{n}}-{\text{d}}_{\text{n}}\right)}^{2}$$

where:

N is number of patterns.

y_n _is output of network

d_n _is desired output.

### fNIRs data acquisition

**Figure 3:**
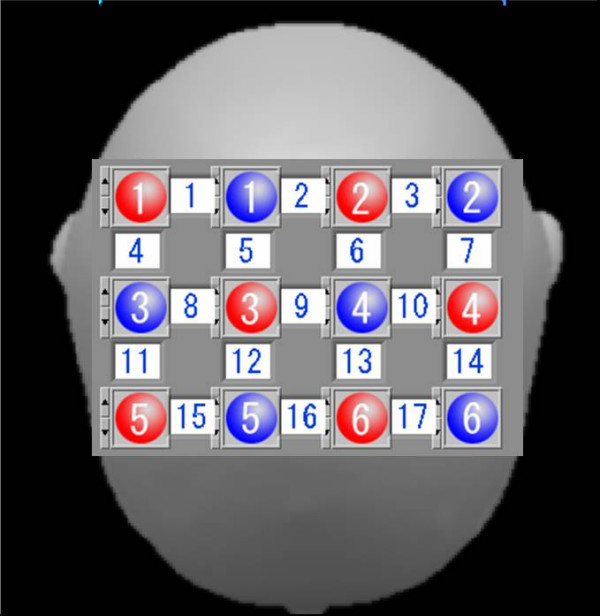
Positions of optodes on the subject's head.

We used a multichannel fNIRs instrument, OMM-3000 from Shimadzu Corporation, Japan, for acquiring oxygenated hemoglobin and deoxygenated hemoglobin concentration changes. The system operated at three different wavelengths of 780 nm, 805 nm and 830 nm, emitting an average power of 3 mW.mm^-2^. The illuminator and detector optodes were placed on the scalp. The detector optodes were fixed at a distance of 3 cm from the illuminator optodes. The optodes were arranged above the hemisphere on the subject's head as shown in Fig. 3.

Near-infrared rays leave each illuminator, pass through the skull and the brain tissue of the cortex and are received by the detector optodes. The photomultiplier cycles through all the illuminator-detector pairings to acquire data at every sampling period. The data were digitized by the 16-bit analog to digital converter.

Because oxygenated and deoxygenated hemoglobin have characteristic optical properties in the visible and near-infrared light range, the change in concentration of these molecules during neurovascular coupling can be measured using optical methods By measuring absorption changes at two (or more) wavelengths, one of which is more sensitive to oxy-Hb, the other to deoxy-Hb, changes in the relative concentrations of these chromophores can be calculated. Using these principles, researchers have demonstrated that it is possible to assess brain activity through the intact skull in adult humans [3].

The fNIRs instrument was capable of storing the raw signals for each of channels, one of which consists of the intensity values of 3 wavelengths, as well as the derived values of oxygenated hemoglobin [Ox-Hb], deoxygenated hemoglobin [Deox-Hb] and total hemoglobin [total-Hb]= [Ox-Hb] + [Deox-Hb] concentration changes for all time points in an output file in a pre-specified format. Under the view of recognition brain activities, we chose the total hemoglobin [total-Hb] concentration changes to analysis its functions.

In this work, we investigate 2 tests to recognition brain activities. Test 1 is implemented with a 32 year old male doing three tasks, as follow:

Task 1: controlling physical motion of right arm,

Task 2: imagining the motion of right arm,

Task 3: relaxing.

Each of tasks is measured during 3 minutes, by 7 channels, and sampling frequency 18 Hz.

**Figure 4:**
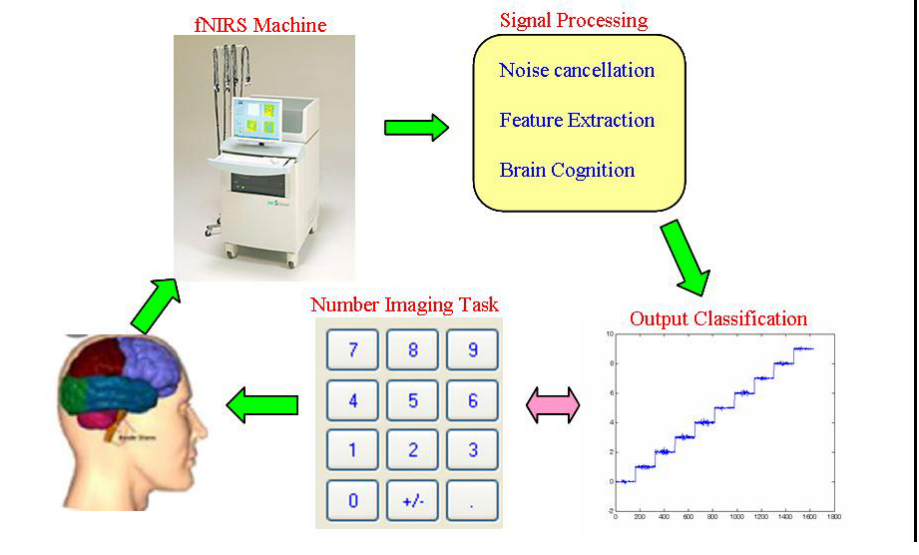
An experiment recognizing brain activities imagining numerical push on a calculator, outputs from 0 to 9 corresponding to pushing numbers from 0 to 9.

Test 2 is implemented with a 28 year old male with mission imagining numerical push on a calculator as Fig. 4. Each of imagining tasks corresponding to a number is measured during 1 minute, by 17 channels, and sampling frequency 10 Hz.

## Results and Discussion

### Fractal Dimension

**Figure 5:**
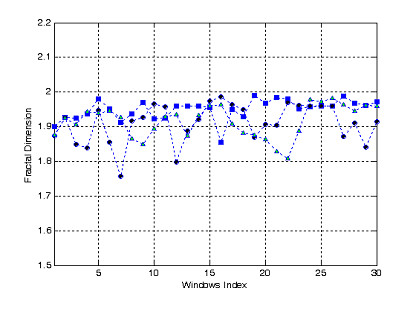
Fractal dimension of three fNIRs channels of 3 tasks of the first test corresponding to circle-point line, square-point line, and triangle-point line.

The first test acquired 3275 points, in which each 100 point is enough to calculate fractal dimension, D_f_, called window index runing along the signals. Computing results are shown in Fig. 5.

**Figure 6:**
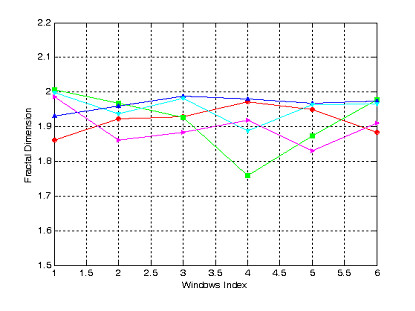
Fractal dimension of 5 fNIRs channels of second imagining test corresponding to pushing number from 0 to 4.

The second test acquired 600 points, in which each 100 point is enough to calculate fractal dimension, D_f_, called window index runing along the signals. Computing results are shown in Fig. 6.

Fig. 5 and Fig. 6 shown that fractal dimension mostly more over than 1.9 indicating high degree of complexity of fNIRs signals as well as complexity of brain dynamics generating the given bio-signals. However these computing results can not demonstrate difference in each task of human brain activities. Therefore, we use model combining Wavelet Transform and Neural Networks to recognizing brain activities.

### Recognition by Wavelet-Neuron Model

In the first test, Wavelet mother is chosen discrete approximation of Meyer wavelet. Level of wavelets decomposition, j, is 3. SNRgain are calculated for each channel and shown in Table 1.

**Table 1:** Signal to noise ratio *(SNR) *gain of 7 channels and 3 tasks

Total-Hb	Ch-1	Ch-2	Ch-3	Ch-4	Ch-5	Ch-6	Ch-7
Task 1	2.7073	2.5748	3.1227	2.8987	0.25172	1.263	0.88898
Task 2	4.7043	1.1575	5.2625	2.2889	0.70114	0.75794	1.1248
Task 3	3.0046	2.2346	3.5706	2.9905	0.30904	1.4218	0.91027

**Figure 7:**
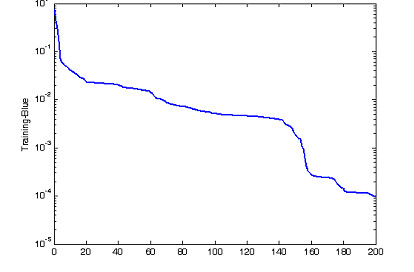
The error of neural network training processing corresponding to 200 epochs of the first experiment.

**Figure 8:**
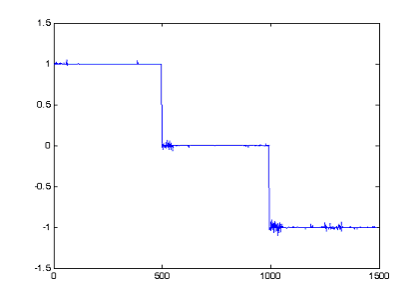
Output of neural network recognizing 3 distinguished tasks of brain activities.

From Table 1, SNR-gain average is calcutated as SNRgain-average = 2.10 Multilayer neural network is built with 3 layers. Input layer consists of 7 neurons corresponding to 7 fNIRs channels. 7 neurons are set for hidden layer and 1 neuron for output layer. The transfer functions of the hidden layer are chosen tagsig-function while the transfer functions of output neurons are purelin-function, a linear function, for representation of many different classes, output equals to +1, 0, -1 corresponding to task 1, 2, 3. The error of Neural training processing shows in Fig. 7, with mean square error of classification is 9.82e-05 in 200 epochs. The output of neural model indicates separately 3 distinguished tasks in Fig. 8.

**Figure 9:**
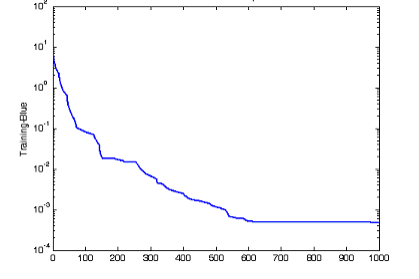
The error of neural network training processing corresponding to 1000 epochs of the second experiment.

**Figure 10:**
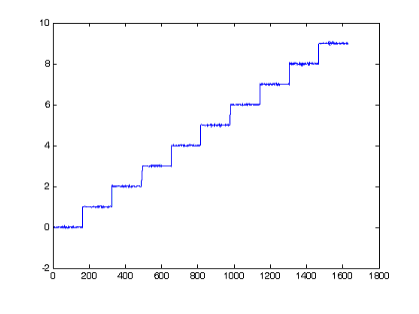
Output of neural network recognizing 10 distinguished tasks of brain activities.

In the second test, SNG-gain average is calculated as SNRgain-average = 2.97. Multilayer neural network is built with 3 layers. Input layer consists of 17 neurons corresponding to 17 channels of fNIRs signals. 17 neurons are set for hidden layer and 1 neuron for output layer. The transfer functions of the hidden layer are chosen tagsig-function while the transfer functions of output neurons are purelin-function, a linear function, for representation of many different classes, output values from 0 to 9 correspondind to numerical imagining from 0 to 9. The error of Neural training processing shows in Fig. 9, with mean square error of classification is 4.79e-04 in 1000 epochs. The output of neural model indicates separately 10 distinguished tasks in Fig. 10.

All two experiments show that classified wavelet-neuron models obtain the high accuracy. The results determine advantages of wavelets analysis as preprocessing and neural networks as classified models.

With many advantages of fNIRs, safe, portable, affordable and high accuracy of computing pattern recognition. A Brain-computer interface (BCI) using fNIRs signals will be developed as an alternate mode of communication and environmental control. Especially disable patients with cognitive ability to communicate with their social environment can live with a reasonable quality of life over extended period time.

## Conclusion

In this study, we have demonstrated the feasibility of fNIRs analysis to recognize human brain activities. fNIRs opens many excellent opportunities to cognition brain activities and interface to computer as future BCIs. The limited paper contributes analyzing nonlinear characteristics of fNIRs by Higuchi's fractal dimension, extracting signal features by wavelet transforms, and recognizing brain activities by neural network. In future, we will indicate the potential use of such techniques to online fNIRs-BCI systems.

## Authors' contributions

All authors contributed equally to this work as well as read and approved the final manuscript.
